# A minimum valency of 4 is required for robust activation of platelets in flow cytometry by multivalent nanobodies to Glycoprotein VI, C-type lectin-like receptor 2 and Platelet Endothelial Aggregation Receptor 1

**DOI:** 10.1016/j.rpth.2025.103196

**Published:** 2025-09-24

**Authors:** Rachel E. Lamerton, Eleyna M. Martin, Jacqueline Perry, Adam F. Cunningham, Steve P. Watson, Andrew L. Frelinger

**Affiliations:** 1 Department of Cardiovascular Sciences, College of Medicine and Health, University of Birmingham, Birmingham, UK; 2 Department of Immunology and Immunotherapy, College of Medicine and Health, University of Birmingham, Birmingham, UK; 3 Center for Platelet Research Studies, Dana-Farber/Boston Children’s Cancer and Blood Disorders Center, Harvard Medical School, Boston, Massachusetts, USA

**Keywords:** ligands, flow cytometry, platelet activation, receptors, cell surface, signal transduction

## Abstract

**Background:**

We have reported that trivalent and tetravalent nanobodies against glycoprotein (GP)VI, C-type lectin-like receptor (CLEC)-2, and platelet endothelial aggregation receptor (PEAR)1 stimulate powerful aggregation and adenosine triphosphate secretion in human platelets.

**Objectives:**

This study aimed to evaluate changes in platelet surface GPs elicited by activation of GPVI, CLEC-2, and PEAR1 using trivalent and tetravalent nanobodies.

**Methods:**

The effect of the crosslinked nanobodies on P-selectin was measured in whole blood and washed platelets with and without secondary mediator inhibitors using classical flow cytometry and on 16 platelet surface GPs in whole blood using multispectral flow cytometry.

**Results:**

Trivalent nanobodies to GPVI and CLEC-2 stimulated modest (<60% of collagen-related peptide) expression of P-selectin in whole blood (10-fold dilution) and washed platelets (2 × 10^7^ mL), whereas tetravalent nanobodies induced a response approaching that of collagen-related peptide. Stimulation of P-selectin expression was partially reduced by inhibitors of adenosine diphosphate (ADP) and thromboxane A_2_, indicating secondary platelet activation despite the low platelet concentration. By multispectral flow cytometry, tetravalent nanobodies to GPVI and CLEC-2 stimulated similar maximal fibrinogen binding and platelet surface α-granule (TLT-1 and CD154) and δ-granule (CD63) markers, but lower levels of the lysosomal marker CD107a. The tetravalent PEAR1 nanobody showed partial agonist activity in some donors but full activity in others.

**Conclusion:**

Tetravalent nanobodies to GPVI and CLEC-2 stimulate powerful activation of platelets at low nanomolar concentrations in flow cytometry. In contrast, trivalent nanobodies are partial agonists. The defined stoichiometry of the nanobodies will aid development of standardized platelet flow cytometry assays.

## Essentials

• We previously showed that trivalent and tetravalent nanobodies to GPVI, CLEC-2 and PEAR1 stimulate powerful platelet aggregation and secretion as measured using LTA.
• To investigate the minimum valency required for robust activation of each receptor we measured changes in multiple activation markers using classical and multispectral flow cytometry.
• Tetravalent nanobodies to GPVI and CLEC-2, more than trivalent nanobodies, induced consistent, high, platelet surface expression of α- δ- and lysosomal granule markers and corresponding populations of highly activated platelets across donors.
• Activation with tetravalent PEAR1 nanobodies was less robust, varied between donors, and produced fewer highly activated platelets.
• The defined stoichiometry of the nanobodies will aid development of standardised platelet flow cytometry assays which may be useful for assessing patients with platelet function disorders.

## 1 Introduction

Platelets are involved in the maintenance of hemostasis and other pathways including inflammation and host defence. The immune receptors—glycoprotein (GP)VI, C-type lectin-like receptor (CLEC)-2 [1,2] and platelet endothelial aggregation receptor (PEAR)1 [3]—have a minimal role in haemostasis but are implicated in many of these wider roles.

GPVI and CLEC-2 signal via tyrosine-based motifs in their cytosolic tails as a result of clustering by multivalent ligands [1]. GPVI signals via Src and Syk tyrosine kinases through its associated Fc receptor γ-chain homodimer via an immunoreceptor tyrosine-based activation motif (ITAM) characterized by 2 YxxLs [4]. GPVI is a receptor for collagen and the synthetic derivative collagen-related peptide (CRP), which is composed of a repeated glycine–proline–hydroxyproline tripeptide motif [5]. Both ligands have an unknown stoichiometry [6,7] and exhibit batch variation [8]. CLEC-2 also signals through Src and Syk kinases but via a single YxxL motif known as a hemITAM [9]. Podoplanin is the endogenous ligand for CLEC-2, while snake venom toxin rhodocytin is a CLEC-2 ligand widely used in functional studies. Rhodocytin is an (αβ)_2_ tetramer that can also form higher-order multimers and is polydisperse [[10], [11], [12]].

PEAR1 signals through a Src and phosphoinositide 3-kinase–driven pathway, leading to activation of Akt [[13], [14], [15]]. PEAR1 has a single YxxM in its cytosolic tail, with phosphoinositide 3-kinase crosslinking 2 phosphorylated motifs through its tandem SH2 domains [15]. In contrast to ITAM receptors, signaling of PEAR1 is independent of Syk [15]. It is activated by sulfated polysaccharides such as fucoidans and heparin, but its endogenous ligand is not known [3,14]. The polysaccharides show significant batch variation and are heterogeneous in structure [15].

We have developed multivalent nanobodies of known valency and have reported that trivalent and tetravalent nanobodies stimulate powerful aggregation and dense granule secretion of platelets at nanomolar concentrations that resemble the responses to widely used ligands, including CRP and rhodocytin [16,17]. The multivalent nanobodies are the first ligands of known stoichiometry for these receptors. This is important as this class of receptors are activated through clustering, with the response dependent on ligand valency and receptor density [18].

Flow cytometry is widely used in research and clinical laboratories with the advantage of requiring small sample volumes. However, several of the commonly used ligands for the GP receptors cannot be used in flow cytometry, including collagen due to its fibrillar nature and podoplanin, which is a transmembrane protein. These restrictions do not apply to the multivalent nanobodies. The developments in multispectral flow cytometry enable the measurement of a much higher number of cell markers than traditional flow cytometry, providing greater insight into molecular changes occurring following platelet activation. Several groups have used multispectral flow cytometry to measure up to 16 surface markers in platelets using minimal amounts of blood [19,20].

In the present study, we compared the ability of the crosslinked GPVI, CLEC-2, and PEAR1 nanobodies of known valency to stimulate platelet activation using classical and multispectral flow cytometry. The results showed that trivalent ligands, which we previously showed are sufficient to stimulate full platelet aggregation [16], stimulate weak expression of α- and δ-granule markers but that the response to the tetravalent ligands approaches that of CRP. The trivalent and tetravalent nanobodies should therefore be classified as partial agonists with the response proportionate to the number of epitopes. The magnitude of the response to the PEAR1 nanobody shows a wider variation between donors relative to the GPVI and CLEC-2 nanobodies.

## 2 Methods

### 2.1 Ethical approval

This experimental work conformed to the Declaration of Helsinki, and research ethics for the collection of blood from healthy consenting volunteers was granted by the University of Birmingham Internal Ethical Review panel (ERN_11-0175-AP5) and Boston Children’s Hospital Institutional Review Board (protocol X09-09-0503). The donors were aged between 18 and 71 years, with similar numbers of males and females. The donors had not fasted prior to donation and had not taken drugs that interfere with platelet activation in the previous 10 days. The platelet count of the donors was in the normal range (1.5-4.5 × 10^8^/mL).

### 2.2 Materials

Prostacyclin was purchased from Caymen Chemicals. Indomethacin, ticagrelor, and bovine serum albumin (BSA; A4697) were purchased from Merck. For standard flow cytometry, allophycocyanin mouse antihuman CD62P (clone AK4), allophycocyanin mouse immunoglobulin G1 k isotype control (clone MOPC21) and FITC mouse antihuman CD41 (clone HIP8), all from Biolegend, were used. Collagen-related peptide (CRP) was purchased from CambCol. Phe-Pro-Arg-chloromethylketone (PPACK) was purchased from Cambridge Biosciences and Gly-Pro-Arg-Pro (GPRP) from MedChemExpress. Vendors and catalog numbers for antibodies used in multispectral flow cytometry are described in Supplementary Table.

### 2.3 Generation of trivalent and tetravalent nanobodies

Trivalent and tetravalent nanobodies to GPVI and CLEC-2 and tetravalent nanobody to PEAR1 were generated as described previously [16]. In brief, nanobodies targeted against the immunoglobulin, C-type lectin-like domains, and EGF-like repeat 12-13 of GPVI, CLEC-2, and PEAR1, respectively, were generated in collaboration with Vlaams Instituut voor Biotechnologie Nanobody Core [15,16,21]. The most potent nanobodies to GPVI and CLEC-2, namely Nb2 and LUAS, respectively, were crosslinked using a flexible (GGGGS)3 linker between 3 copies of the original nanobody sequence to generate trivalent ligands. Tetravalent nanobodies, including to PEAR1 using nanobody Nb138, were generated using a (GGGGS)3 linker between 2 copies of the original nanobody sequence, followed by a mouse Fc domain (immunoglobulin G2a) at the C-terminus. These are named Nb2-3 and LUAS-3 for the trivalent nanobodies and Nb2-2-Fc, LUAS-2-Fc and Nb138-2-Fc for the tetravalent nanobodies. The trivalent and tetravalent nanobodies were expressed in mammalian HEK293T cells. Trivalent nanobody constructs contained a C-terminal cleavable his6-tag, which allowed purification by nickel affinity chromatography, while the tetravalent nanobodies were purified by protein G affinity chromatography. Purity was assessed by sodium dodecyl sulfate–polyacrylamide gel electrophoresis, as described in the study by Martin et al. [16]. The concentration of purified nanobody was determined using a NanoDrop spectrophotometer (ND-1000; Geneflow), measuring absorbance at 280 nm according to the manufacturer’s protocol. Purified nanobodies were stored at −70 °C.

### 2.4 Flow cytometry measurements of P-selectin in whole blood and washed platelets

Blood from volunteers who had not taken aspirin or other nonsteroidal antiinflammatory agents for 10 days was collected in 3.2% sodium citrate VACUETTE blood containers (Greiner Bio-One) using a 21-gauge needle.

Washed platelets were prepared as previously described [16] and resuspended in modified Tyrode buffer (129 mM NaCl, 0.34 mM Na_2_HPO_4_, 2.9 mM KCl, 12 mM NaHCO_3_, 20 mM HEPES, 1 mM MgCl_2_, and 5 mM glucose; pH 7.3) at a concentration of 2 × 10^7^ cells/mL. Whole blood was diluted 1 in 10 with modified Tyrodes containing PPACK (75 μM), GPRP (2.5 mM) and 0.05% BSA, followed by the addition of 2.5 mM Ca^2+^ after a 5-minute incubation.

Ticagrelor (10 μM) and indomethacin (1 μM) or vehicle (dimethylsulfoxide [final concentration 0.005%] or phosphate-buffered saline) control were added to whole blood and washed platelets and incubated for 5 minutes. Experiments were performed at room temperature in a 96-well plate, using 45 μL of whole blood or washed platelets (2 x 10^7^/mL). Nanobodies and CRP were prepared at 10× final concentration with 5 μL added to blood or platelets to give final concentrations of 3 μg/mL CRP and 0.03 to 30 nM nanobody, before being incubated at room temperature for 30 minutes. P-selectin antibody or isotype control were added at a 1:40 dilution and samples incubated in the dark for 20 minutes at room temperature. Samples were diluted 5 times in phosphate-buffered saline before analyzing at least 10,000 platelet events using a BD Accuri C6 Plus.

### 2.5 Multispectral flow cytometry

Multispectral flow cytometry was performed as previously described [22,23]. In brief, assay tubes were prepared by combining 15 μL antibody cocktail (containing all antibodies in Supplementary Table, except GPVI and CD36), 9 μL assay buffer (final concentrations: 2.5 mM CaCl_2_, 75 μM PPACK and 2.5 mM GPRP, 10 mM HEPES-Tyrodes buffer with 0.05% BSA), and 3 μL of 10× agonist (eg, nanobodies, ADP, and thrombin receptor activating peptide). To this mixture, 3 μL citrate-anticoagulated whole blood was added and mixed by gentle titration. Antibodies were allowed to bind to surface exposed receptors on intact cells for 30 minutes at room temperature. Samples were then fixed with 500 μL lyse/fix solution (BD Biosciences) supplemented with 1 mM CaCl_2_. Fixed samples were centrifuged, and the pellet resuspended in assay buffer with GPVI and CD36 antibodies and incubated an additional 30 minutes at room temperature and then diluted with 250 μL BD fix/lyse with 1 mM CaCl_2_. Samples were analyzed on a 5-laser Cytek Aurora with thresholds on side-scatter and CD61 fluorescence (R4). Forward and side-light scatter was used to gate for singlets, and then CD61 and CD42a staining was used to identify platelets. At least 10,000 CD61^+^ events were collected. Unmixing was performed using single-stain platelet controls in Cytek SpectroFlo software, version 3.3. High-dimensional data analysis to identify platelet subsets was performed using FAUST (full annotation using shaped-constrained trees) [24] implemented in the online data analysis platform Tercen (https://www.tercen.com). Trajectory analysis using Potential of Heat-diffusion for Affinity-based Trajectory Embedding [25] was used to infer the sequence of exposure of activation markers and the order of appearance of platelet subpopulations. Potential of Heat-diffusion for Affinity-based Trajectory Embedding analyses were performed in OMIQ (https://app.omiq.ai/) using all markers and default parameters (2 components, 5 nearest neighbors, and Euclidean KNN distance metric).

### 2.6 Statistical analysis

Data were analyzed and presented using GraphPad Prism 10.3.1 software. Curves were fitted with nonlinear regression ([agonist] vs response—variable slope) from which half-maximal effective concentration (EC_50_) values were obtained. Bars represent SEM.

## 3 Results

### 3.1 Trivalent and tetravalent nanobody ligands stimulate P-selectin expression in washed platelets and whole blood

**Figure 1:**
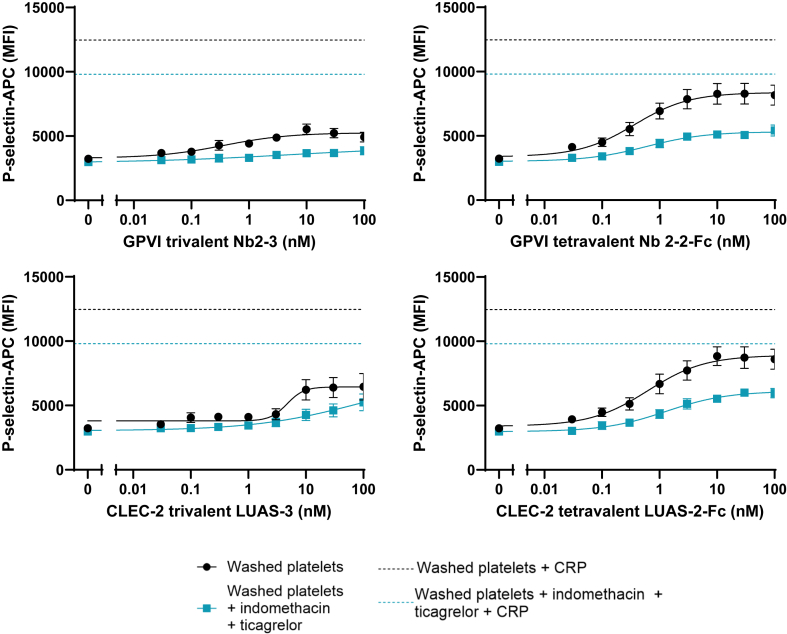
Tetravalent nanobodies against glycolprotein (GP)VI (Nb2-2-Fc) and C-type lectin-like receptor (CLEC)-2 (LUAS-2-Fc) stimulate greater platelet activation responses than trivalent nanobodies in washed platelets. Washed platelets at 2 × 10^7^/mL were incubated either with 1 μM indomethacin and 10 μM ticagrelor or vehicle control, before being stimulated with the nanobody agonist for 30 minutes. P-selectin expression was assessed using flow cytometry. Collagen-related peptide (CRP) at 3 μg/mL was used as a positive control—dashed lines show average response. Results are expressed as median fluorescence intensity (MFI) of P-selectin antibody. Curves were fitted with nonlinear regression ([agonist] vs response—variable slope), as shown in solid lines; *n* ≥6; bars represent SEM. MFI, median fluorescence intensity. EC_50_ values: Nb2-3 0.36, nM; Nb2-2-Fc, 0.37 nM; LUAS-3, 4.69 nM; LUAS-2-Fc, 0.62 nM.

The dose–response relationships for the trivalent and tetravalent nanobodies to GPVI and CLEC-2 for expression of the α-granule protein, P-selectin, were determined in washed platelets (2 × 10^7^/mL) and compared with a maximally effective concentration of CRP (3 μg/mL) (Figure 1). Supplementary Figure 1 shows individual CRP responses. Maximal concentrations of the trivalent nanobodies, Nb2-3 (GPVI) and LUAS-3 (CLEC-2), stimulated <50% of the response to CRP. Nevertheless, both nanobodies were potent, with EC_50_ values of 0.36 and 4.69 nM, respectively, with the potency of LUAS-3 consistent with the lower affinity of the parent nanobody, LUAS, relative to Nb2 [16]. The tetravalent nanobodies stimulated a larger response, which approached 75% of that to CRP. The EC_50_ values for the 2 tetravalent nanobodies, Nb2-2-Fc and LUAS-2-Fc, were 0.37 and 0.62 nM, respectively. The increase in affinity of LUAS-2-Fc relative to LUAS-3 is due to avidity as reported previously [16]. These studies were repeated in the same donors in the presence of inhibitors of the major feedback mediators, ADP and thromboxane A_2_, namely ticagrelor and indomethacin, respectively. The maximal response to the trivalent and tetravalent nanobodies, and to CRP, was reduced by up to 40% in the combined presence of the inhibitors, as shown in Figure 1. This demonstrates that secondary feedback agonists contribute to platelet activation despite the approximate 10-fold reduction in platelet count relative to that in whole blood.

**Figure 2:**
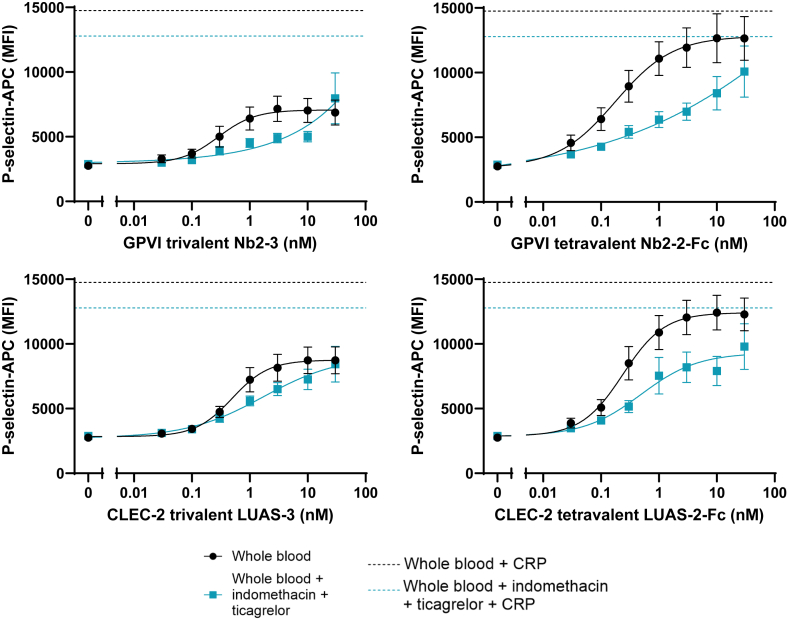
Tetravalent nanobodies against glycolprotein (GP)VI (Nb2-2-Fc) and C-type lectin-like receptor (CLEC)-2 (LUAS-2-Fc) stimulate greater platelet activation responses than trivalent nanobodies in whole blood. Whole blood diluted 1 in 10 was incubated either with 1 μM indomethacin and 10 μM ticagrelor or vehicle control, before stimulation with the nanobody agonist for 30 minutes. P-selectin expression was assessed using flow cytometry. Collagen-related peptide (CRP) at 3 μg/mL was used as a positive control—dashed lines show average response. Curves were fitted with nonlinear regression ([agonist] vs response—variable slope), as shown in solid lines; *n* ≥6; bars represent SEM. EC50 values: Nb2-3, 0.29 nM; LUAS-3, 0.49 nM; Nb2-2-Fc, 0.18 nM; and LUAS-2-Fc, 0.24 nM.

To assess whether the presence of other blood cells and/or plasma affected the platelet responses to the nanobodies, we repeated the abovementioned studies in whole blood diluted 1:10 in HEPES-Tyrodes buffer (Figure 2). The platelet concentrations used in these studies were in the range of 1.5 to 4.0 × 10^7^/mL, reflecting the variation in platelet count between donors. The trivalent and tetravalent nanobodies stimulated a greater maximal response to that in washed platelets and with slightly lower EC_50_ values (Figure 2). As with the result in washed platelets, the response to the trivalent and tetravalent nanobodies were reduced in the presence of ticagrelor and indomethacin.

### 3.2 Immunophenotypes of GPVI and CLEC-2 nanobody–stimulated platelets by multispectral flow cytometry

We have shown that classical flow cytometry can be used to measure P-selectin levels on platelets activated by nanobodies to GPVI and CLEC-2. However, GPVI and CLEC-2 activation alters the expression of many more platelet surface molecules, potentially generating platelets with unique expression profiles (immunophenotypes). To investigate these changes, we used multispectral flow cytometry to measure the levels of 16 surface markers.

**Figure 3:**
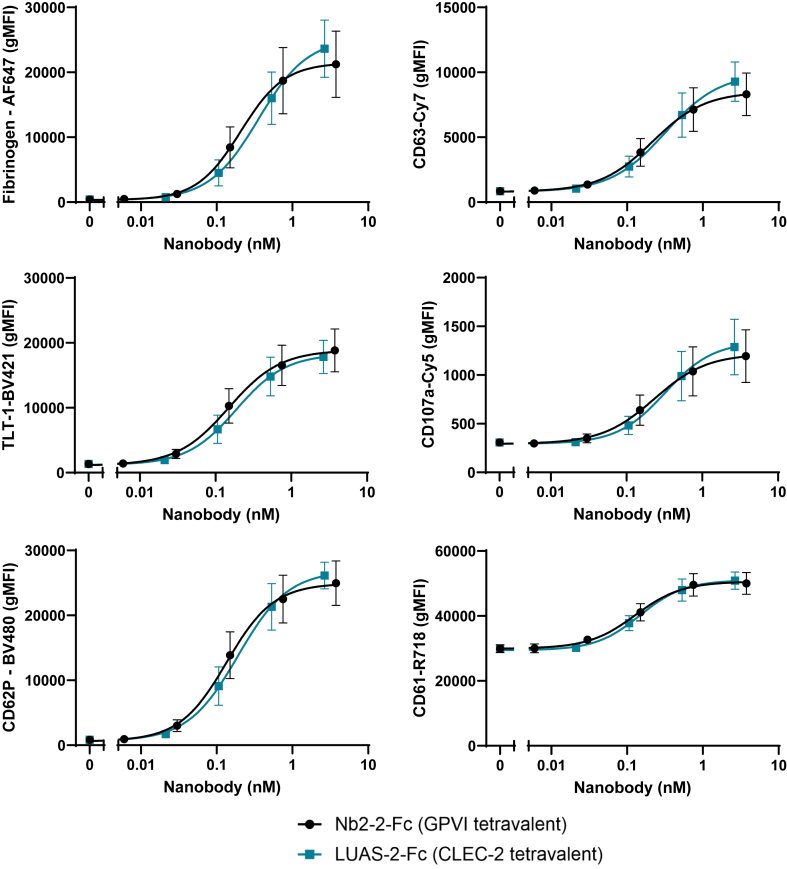
Multispectral flow cytometry can be used to assess multiple platelet markers in response to tetravalent nanobodies. Whole blood was diluted 1 in 10 in antibody cocktail and agonist and incubated for 30 minutes. Data are expressed as geometric mean fluorescence intensity (gMFI). Curves were fitted with nonlinear regressions ([agonist] vs response—variable slope) as shown by solid lines; *n* = 6; bars represent SEM. Black circles, glycoprotein VI tetravalent nanobody—Nb2-2-Fc; turquoise squares, C-type lectin-like receptor 2 tetravalent nanobody—LUAS-2-Fc.

As shown in Figure 3, the activation markers fibrinogen (Fg), CD63, TLT-1, and CD107a increased with increasing concentrations of the tetravalent nanobodies against GPVI and CLEC-2 (Nb2-2-Fc and LUAS-2-Fc, respectively). The inclusion of CD62P corroborated our results from Figure 2. Other proteins whose levels increased are CD61, CD191, C3b, annexin V (Ann V), TLR9, and CD154 (Supplementary Figure 2). The constitutive platelet membrane proteins GPVI, CD31, CD42a, CD32, and CD36 showed relatively little change in expression as expected (Supplementary Figure 3). The responses to the 2 tetravalent nanobodies against GPVI and CLEC-2 were similar.

### 3.3 Response to tetravalent nanobody activation of PEAR1 is donor dependent

**Figure 4:**
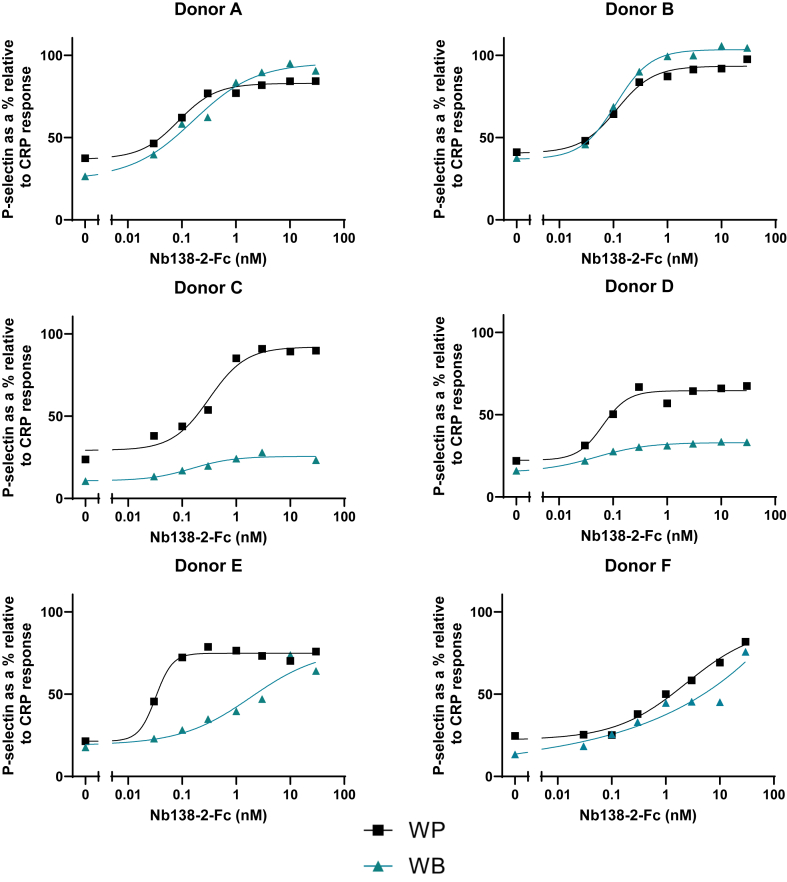
P-selectin responses to the tetravalent platelet endothelial aggregation receptor (PEAR)1 nanobody (Nb138-2-Fc) vary between donors. Donor P-selectin responses to the PEAR1 tetravalent nanobody are expressed as a percentage relative to their P-selectin response to 3 μg/mL collagen-related peptide (CRP). Symbols show individual data points, solid lines show nonlinear regression curve fitting. Black squares, washed platelets (WPs); turquoise triangles, whole blood (WB).

In contrast to the reproducible effects induced by the tetravalent nanobodies against GPVI and CLEC-2, the maximal increase in P-selectin in response to the tetravalent nanobody for PEAR1, Nb138-2-Fc showed a wide variation between donors relative to CRP (Figure 4). The maximal responses in washed platelets varied between 65% and 93%, while in whole blood, it varied between 25% and 102% of that to CRP. The EC_50_ ranged from 0.03 to 2.38 nM in washed platelets and 0.05 to 1.88 nM in whole blood.

**Figure 5:**
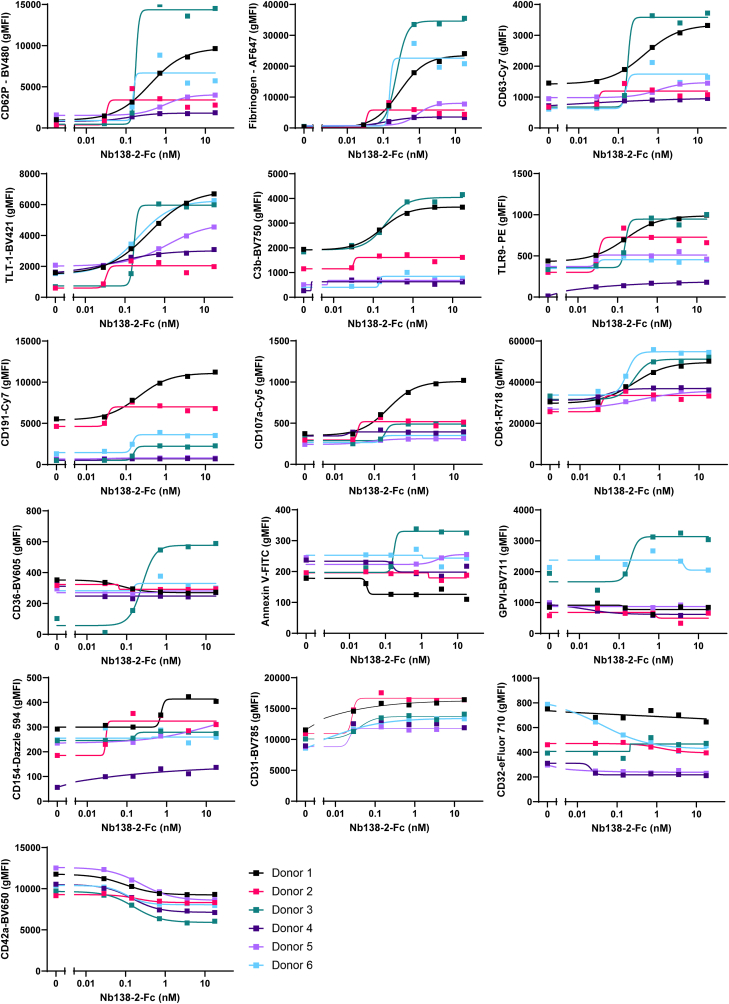
Donor variation to the tetravalent platelet endothelial aggregation receptor (PEAR)-1 nanobody (Nb138-2-Fc) is confirmed with multispectral flow cytometry. Whole blood was diluted 1 in 10 in antibody cocktail and Nb138-2-Fc and incubated for 30 minutes. Curves were fitted with nonlinear regressions ([agonist] vs response—variable slope); *n* = 6; bars represent SEM. Donors are represented by the same color in each graph as per the key.

To explore the donor variation further, we used the multispectral flow cytometry panel. Donor variation in responses to PEAR1 tetravalent nanobody, Nb138-2-Fc, was seen across nearly all of the platelet activation markers studied (Figure 5). For example, donor 3 showed the highest activation levels in response to Nb138-2-Fc across CD62P, fibrinogen, CD63, C3b, CD36, Ann V, and GPVI. Donor 1 also showed strong activation to most of these markers, but they also showed strong responses with CD191 and CD107a, levels of which did not increase in donor 1. In contrast, donors 2 and 4 showed very little response to Nb138-2-Fc across any of the markers (despite responding to the GPVI and CLEC-2 tetravalent nanobodies).

### 3.4 Identification of platelet subtypes by machine learning high-dimensional analysis

**Figure 6:**
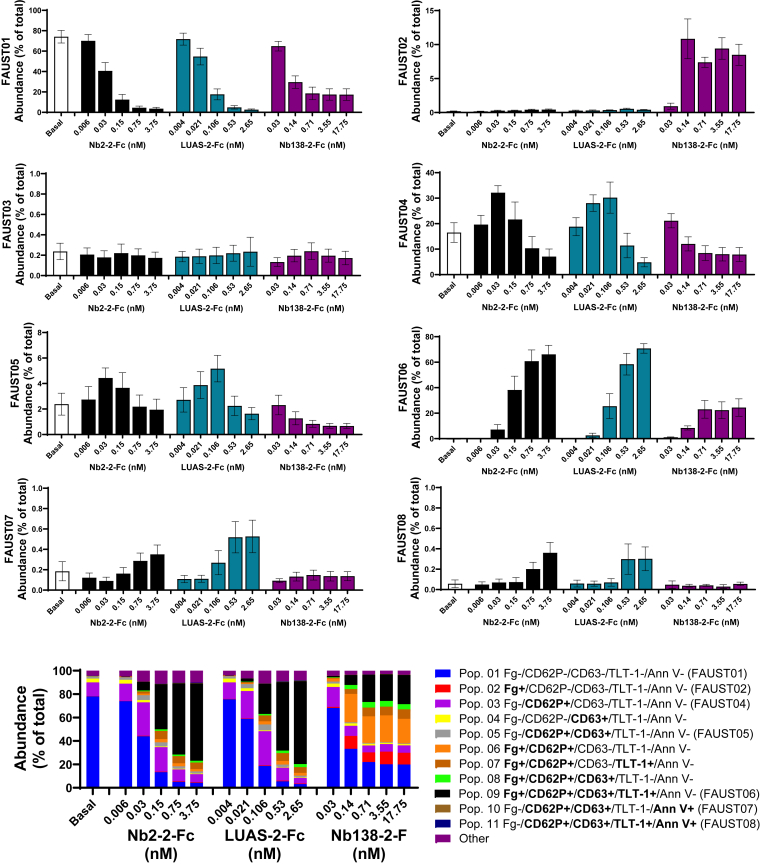
Full annotation using shaped-constrained trees (FAUST) analysis identifies distinct platelet subpopulations in response to platelet endothelial aggregation receptor (PEAR)1 (Nb138-2-Fc) tetravalent nanobody compared with glycoprotein (GP)VI (Nb2-2-Fc) and C-type lectin-like receptor (CLEC)-2 (LUAS-2-Fc) tetravalent nanobodies. FAUST machine learning high-dimensional analysis was performed using the data obtained from multispectral flow cytometry. On the basis of 5 markers of activation, 8 distinct platelet subpopulations were identified. Bottom graph, further population analysis was carried out to identify the major subpopulations not labeled by FAUST (see also Supplementary Figure 4); *n* = 6; bars represent mean ± SEM.

FAUST machine learning high-dimensional analysis [24] was performed on the multispectral flow cytometry data to identify platelet subsets common to all agonists. Eight distinct platelet populations were identified based on 5 markers of activation (Figure 6). As expected, the majority of unstimulated platelets fell into the FAUST01 population, which was negative for CD62P, TLT-1, CD63, Fg, and Ann V. Upon stimulation with the nanobodies, changes in the abundance of the FAUST subpopulations suggested the sequence of expression of surface markers triggered by increasing levels of nanobody. For example, at 0.03 nM Nb2-2-Fc, the level of FAUST01 platelets reduced from 70% to 40%, and the levels of FAUST04 (CD62P^+^, TLT-1^−^, CD63^−^, Fg^−^, Ann V^−^) and FAUST05 (CD62P^+^, CD63^+^, TLT-1^−^, Fg^−^, Ann V^−^) increased, showing partially activated subsets of platelets, with few in the fully activated FAUST06 (CD62P^+^, TLT-1^+^, CD63^+^, Fg^+^, Ann V^−^) population. In contrast, with the higher dose of Nb2-2-Fc, there are fewer platelets in the FAUST04 and FAUST05 populations, with over 65% falling into the FAUST06 category. Levels of FAUST02 (Fg^+^ only) increased slightly with increasing nanobody treatment, as do those of FAUST07 (CD62P^+^, CD63^+^, Ann V^+^, TLT-1^−^, Fg^−^) and FAUST08 (CD62P^+^, TLT-1^+^, CD63^+^, Ann V^+^, Fg^−^), but overall, these populations accounted for less than 2% of platelets. The only population to remain the same regardless of nanobody concentration was FAUST03, which being Ann V^+^ only, indicating that these are unresponsive platelets that are present in the circulation.

There were several differences in the platelet subpopulations observed with FAUST analysis for the PEAR1 tetravalent nanobody compared with those for the tetravalent GPVI and CLEC-2 (Figure 6). A striking difference was seen in the FAUST02 (Fg^+^ only) population (Figure 6), indicating activation of the integrin GPIIb-IIIa in the absence of secretion (no exposure of P-selectin, TLT-1, CD63, or CD107a). The PEAR1 nanobody also did not induce the same level of fully activated platelets, as shown by the lower abundance of the FAUST06 population (25% vs 65%-70%), positive for CD62P, TLT-1, CD63, and Fg. Approximately 20% of the platelets stimulated by Nb2-2-Fc and LUAS-2-Fc and 40% of the platelets stimulated by Nb138-2-Fc did not fall into the major 8 categories identified by the FAUST analysis (Supplementary Figure 4). We therefore carried out further analysis to identify these platelets and observed that, for Nb138-2-Fc, 55% of these (22% of total platelets) being Fg^+^, CD62P^+^, CD63^−^, TLT-1^−^, Ann V^−^, a subpopulation that is minimal with Nb2-2-Fc and LUAS-2-Fc stimulation, consistent with a weaker level of activation (bottom of Figure 6; Supplementary Figure 4). Trajectory analysis also highlighted the differences in the platelet activation process in response to Nb2-2-Fc/LUAS-2-Fc and Nb138-2-Fc (Supplementary Figures 5 and 6). The appearance of Pop. 04 to the left of Pop. 03 was consistent with CD63 exposure upon δ-granule release and the contribution of δ-granule constituents such as ADP to subsequent exposure of CD62P.

## 5 Discussion

In this study, we showed that tetravalent nanobodies to GPVI and CLEC-2, in addition to stimulating robust platelet aggregation [16], induce high platelet surface expression of multiple activation markers including P-selectin, TLT-1, CD40L, CD63, and CD107a, whereas their trivalent counterparts induce much lower levels. We demonstrated that platelets activated with nanobodies to GPVI and CLEC-2 cluster into distinct subpopulations and that changes in the abundance of these subpopulations are useful to infer the sequence of activation marker expression. Platelet activation with tetravalent nanobodies to GPVI and CLEC-2, which share a common signaling mechanism, is consistent across donors, whereas response to the tetravalent nanobody targeting PEAR1, which uses a different signaling mechanism, varies between donors and generates distinct ratios of platelet subpopulations. This difference suggests that factors that contribute to PEAR1 signaling vary across donors, whereas factors that contribute to GPVI and CLEC-2 signaling, which share a common mechanism, are more consistent across donors.

In our previous article, we showed that trivalent and tetravalent nanobodies to GPVI, CLEC-2, and PEAR1 stimulate powerful platelet aggregation and secretion as measured using lumi-light transmission aggregometry in washed platelets or platelet-rich plasma [16]. The results show a high consistency in response between donors with the tetravalent antibodies, showing a slightly greater potency reflecting the extra epitope.

In this study, we used the nanobodies in classical and multispectral flow cytometry in whole blood and washed platelets at a concentration that is approximately 10 times lower than that in lumi-light transmission aggregometry. At this concentration of platelets, the trivalent and tetravalent nanobodies show a reduced level of activation compared with the CRP control, especially in the case of the trivalent nanobodies. We also showed that the secondary mediators ADP and thromboxane A_2_ support the response to the nanobodies and to CRP despite the 10-fold depletion. The effects of the P2Y_12_ and cyclooxygenase inhibitors, ticagrelor and indomethacin, respectively, are less significant in whole blood, reflecting a greater sensitivity of platelets (which diminishes on the preparation of washed platelets) to the multivalent nanobodies or the role of other constituents in blood, including leukocytes, in their response.

Multispectral flow cytometry allows measurement of an array of platelet markers, revealing highly detailed information on the activation of platelets. As expected, well-characterized platelet activation markers such as those released by δ-granule (CD63), α-granules (P-selectin and TLT1), lysosomal markers (CD107a), and GPIIb-IIIa activation (Fg binding) increased with increasing concentrations of the nanobody ligands. In addition, they also induced an increase in TLR9 expression, a receptor important in innate immunity for recognition of pathogen-associated molecular patterns, and C3b, a key component of the complement system. A potential explanation for this increase in C3b is the ability for C3b present in plasma to bind newly exposed P-selectin [26]. The machine learning technique, FAUST analysis, allows identification of platelet subpopulations, capturing the heterogeneity of the platelet population in a way that classical flow cytometry cannot. This approach is particularly revealing when comparing the differences between the GPVI and CLEC-2 nanobodies relative to the PEAR1 nanobodies. Stimulating with the tetravalent PEAR1 nanobody reveals a unique subset of platelets (FAUST02) that are positive for Fg and not for CD62P, TLT-1, or CD63. Furthermore, approximately 40% of the platelets were labeled other in the FAUST analysis, likely a result of the known limitations of the FAUST algorithm [24]. Probing this category further revealed a large Fg^+^, CD62P^+^, CD63^−^, TLT-1^−^, Ann V^−^ population to be present (∼22% of total platelets) at levels approximately 10 times higher than in platelets stimulated with Nb2-2-Fc or LUAS-2-Fc, suggesting weaker activation of platelets at this time point by Nb138-2-Fc. This separation of CD62P and TLT-1 expression further points toward TLT-1 being segregated within the α-granule, within a distinct α-granule population or localized to an as-yet-unidentified compartment as suggested previously [27,28]. The analysis did not pick up combinations of platelets that are positive for Ann V, CD62P, and Fg/CD42a (the combination of markers defining procoagulant platelets [29]), demonstrating that the tetravalent nanobodies on their own cannot induce significant procoagulant activity. Similarly, apoptotic platelets (as defined by the absence of P-selectin and presence of Ann V and CD42a or CD41 [29]) were not picked up in amounts larger than 0.5%.

The use of the tetravalent PEAR1 nanobody also showed a greater array of interdonor variation, relative to the responses to the GPVI and CLEC-2 nanobodies and CRP. Some donors responded to near maximal levels of P-selectin expression in washed platelets and whole blood, whereas some showed minimal responses. This trend was also seen in the multispectral flow cytometry studies. There have been several studies showing that single-nucleotide variations in the *PEAR1* gene are associated with platelet aggregation [[30], [31], [32], [33], [34], [35]], responses to antiplatelet therapies [36], and cardiovascular outcomes [37,38], as well as those showing epigenetic PEAR1 modifications affecting platelet function [39]. Further studies using multispectral flow cytometry may help to further characterize these differences.

One major advantage of flow cytometry is its ability to be used in platelet diagnostics with small volumes of blood and in thrombocytopenic individuals. Flow cytometry is recommended by the Platelet Physiology International Society on Thrombosis and Haemostasis (ISTH) Scientific and Standardization Committee Subcommittee as the first and second steps in diagnosing suspected inherited platelet function disorders [40] and is highly amenable for the diagnostic testing of young children and/or those with thrombocytopenia [41]. However, the lack of agonists of known valency for the platelet GP receptors that form the focus of this study has left a gap in the study of these receptors using flow cytometry. This is further underscored by the batch-to-batch variation in activity of many of their ligands and that several cannot be used in this technique. This includes collagen due to its fibrillar nature and the transmembrane protein podoplanin. The application of multivalent nanobodies to aid in bleeding diagnostics has recently been demonstrated by Zivkovic et al. [42] who described the ability of a different tetravalent GPVI nanobody to that used in the present study to distinguish platelets from patients with an inherited platelet function disorder and healthy controls.

In conclusion, we demonstrated the use of our defined valency GPVI, CLEC-2, and PEAR1 nanobodies in both classical and multispectral flow cytometry. We showed that the tetravalent GPVI and CLEC-2 nanobodies induce stronger platelet activation than the trivalent antibodies and that there is interdonor variation in activation responses to the tetravalent PEAR1 nanobody. It remains to be proven whether the partial agonist activity of the trivalent nanobodies is more suitable to the study of patients with platelet function disorders than the more powerful tetravalent nanobodies.

## References

[1] Rayes J., Watson S.P., Nieswandt B. (2019). Functional significance of the platelet immune receptors GPVI and CLEC-2. J Clin Invest.

[2] Harbi M.H., Smith C.W., Nicolson P.L.R., Watson S.P., Thomas M.R. (2021). Novel antiplatelet strategies targeting GPVI, CLEC-2 and tyrosine kinases. Platelets.

[3] Kardeby C., Damaskinaki F.N., Sun Y., Watson S.P. (2021). Is the endogenous ligand for PEAR1 a proteoglycan: clues from the sea. Platelets.

[4] Haining E.J., Nicolson P.L.R., Onselaer M.-B., Poulter N.S., Rayes J., Thomas M.R. (2019). Platelets.

[5] Cejas M.A., Kinney W.A., Chen C., Leo G.C., Tounge B.A., Vinter J.G. (2007). Collagen-related peptides: self-assembly of short, single strands into a functional biomaterial of micrometer scale. J Am Chem Soc.

[6] Knight C.G., Morton L.F., Onley D.J., Peachey A.R., Ichinohe T., Okuma M. (1999). Collagen–platelet interaction: Gly-Pro-Hyp is uniquely specific for platelet Gp VI and mediates platelet activation by collagen1. Cardiovasc Res.

[7] Morton L.F., Hargreaves P.G., Farndale R.W., Young R.D., Barnes M.J. (1995). Integrin α2β1-independent activation of platelets by simple collagen-like peptides: collagen tertiary (triple-helical) and quaternary (polymeric) structures are sufficient alone for α2β1-independent platelet reactivity. Biochem J.

[8] Jarvis G.E., Atkinson B.T., Snell D.C., Watson S.P. (2002). Distinct roles of GPVI and integrin alpha(2)beta(1) in platelet shape change and aggregation induced by different collagens. Br J Pharmacol.

[9] Martin E.M., Zuidscherwoude M., Moran L.A., Di Y., Garcia A., Watson S.P. (2021). The structure of CLEC-2: mechanisms of dimerization and higher-order clustering. Platelets.

[10] Watson A.A., Eble J.A., O'Callaghan C.A. (2008). Crystal structure of rhodocytin, a ligand for the platelet-activating receptor CLEC-2. Protein Sci.

[11] Hooley E., Papagrigoriou E., Navdaev A., Pandey A.V., Clemetson J.M., Clemetson K.J. (2008). The crystal structure of the platelet activator aggretin reveals a novel (ab)2 dimeric structure. Biochemistry.

[12] Sasaki T., Shirai T., Tsukiji N., Otake S., Tamura S., Ichikawa J. (2018). Functional characterization of recombinant snake venom rhodocytin: rhodocytin mutant blocks CLEC-2/podoplanin-dependent platelet aggregation and lung metastasis. J Thromb Haemost.

[13] Nanda N., Bao M., Lin H., Clauser K., Komuves L., Quertermous T. (2005). Platelet endothelial aggregation receptor 1 (PEAR1), a novel epidermal growth factor repeat-containing transmembrane receptor, participates in platelet contact-induced activation. J Biol Chem.

[14] Kauskot A., Di Michele M., Loyen S., Freson K., Verhamme P., Hoylaerts M.F. (2012). A novel mechanism of sustained platelet alphaIIbbeta3 activation via PEAR1. Blood.

[15] Kardeby C., Evans A., Campos J., Al-Wahaibi A.M., Smith C.W., Slater A. (2023). Heparin and heparin proteoglycan-mimetics activate platelets via PEAR1 and PI3Kbeta. J Thromb Haemost.

[16] Martin E.M., Clark J.C., Montague S.J., Moran L.A., Di Y., Bull L.J. (2024). Trivalent nanobody-based ligands mediate powerful activation of GPVI, CLEC-2, and PEAR1 in human platelets whereas FcgammaRIIA requires a tetravalent ligand. J Thromb Haemost.

[17] Clark J.C., Martin E.M., Moran L.A., Di Y., Wang X., Zuidscherwoude M. (2023). Divalent nanobodies to platelet CLEC-2 can serve as agonists or antagonists. Commun Biol.

[18] Maqsood Z., Clark J.C., Martin E.M., Cheung Y.F.H., Moran L.A., Watson S.E.T. (2022). Experimental validation of computerised models of clustering of platelet glycoprotein receptors that signal via tandem SH2 domain proteins. PLoS Comput Biol.

[19] Vadgama A., Boot J., Dark N., Allan H.E., Mein C.A., Armstrong P.C. (2024). Multiparameter phenotyping of platelets and characterization of the effects of agonists using machine learning. Res Pract Thromb Haemost.

[20] Spurgeon B.E.J., Frelinger A.L. (2022). Comprehensive phenotyping of human platelets by single-cell cytometry. Cytometry A.

[21] Slater A., Di Y., Clark J.C., Jooss N.J., Martin E.M., Alenazy F. (2021). Structural characterization of a novel GPVI-nanobody complex reveals a biologically active domain-swapped GPVI dimer. Blood.

[22] Spurgeon B.E.J., Linden M.D., Michelson A.D., Frelinger A.L. (2021). Immunophenotypic analysis of platelets by flow cytometry. Curr Protoc.

[23] Spurgeon B.E.J., Frelinger A.L. (2023). OMIP-097: high-parameter phenotyping of human platelets by spectral flow cytometry. Cytometry A.

[24] Greene E., Finak G., D’Amico L.A., Bhardwaj N., Church C.D., Morishima C. (2021). New interpretable machine-learning method for single-cell data reveals correlates of clinical response to cancer immunotherapy. Patterns.

[25] Moon KR, Dijk D, Wang Z, Gigante S, Burkhardt DB, Chen WS (2019). Visualizing structure and transitions in high-dimensional biological data. Nat Biotechnol.

[26] Del Conde I., Cruz M.A., Zhang H., Lopez J.A., Afshar-Kharghan V. (2005). Platelet activation leads to activation and propagation of the complement system. J Exp Med.

[27] Barrow A.D., Astoul E., Floto A., Brooke G., Relou I.A.M., Jennings N.S. (2004). Cutting edge: TREM-like transcript-1, a platelet immunoreceptor tyrosine-based inhibition motif encoding costimulatory immunoreceptor that enhances, rather than inhibits, calcium signaling via SHP-21. J Immunol.

[28] Smith C.W., Raslan Z., Parfitt L., Khan A.O., Patel P., Senis Y.A. (2018). TREM-like transcript 1: a more sensitive marker of platelet activation than P-selectin in humans and mice. Blood Adv.

[29] Josefsson E.C., Ramstrom S., Thaler J., Lordkipanidze M., COAGAPO study groups (2023). Consensus report on markers to distinguish procoagulant platelets from apoptotic platelets: communication from the Scientific and Standardization Committee of the ISTH. J Thromb Haemost.

[30] Faraday N., Yanek L.R., Yang X.P., Mathias R., Herrera-Galeano J.E., Suktitipat B. (2011). Identification of a specific intronic PEAR1 gene variant associated with greater platelet aggregability and protein expression. Blood.

[31] Eicher J.D., Xue L., Ben-Shlomo Y., Beswick A.D., Johnson A.D. (2016). Replication and hematological characterization of human platelet reactivity genetic associations in men from the Caerphilly Prospective Study (CaPS). J Thromb Haemost.

[32] Herrera-Galeano J.E., Becker D.M., Wilson A.F., Yanek L.R., Bray P., Vaidya D. (2008). A novel variant in the platelet endothelial aggregation receptor-1 gene is associated with increased platelet aggregability. Arterioscler Thromb Vasc Biol.

[33] Johnson A.D., Yanek L.R., Chen M.-H., Faraday N., Larson M.G., Tofler G. (2010). Genome-wide meta-analyses identifies seven loci associated with platelet aggregation in response to agonists. Nat Gen.

[34] Jones C.I., Bray S., Garner S.F., Stephens J., de Bono B., Angenent W.G.J. (2009). A functional genomics approach reveals novel quantitative trait loci associated with platelet signaling pathways. Blood.

[35] Kim Y., Suktitipat B., Yanek L.R., Faraday N., Wilson A.F., Becker D.M. (2013). Targeted deep resequencing identifies coding variants in the PEAR1 gene that play a role in platelet aggregation. PLoS One.

[36] Lewis J.P., Riaz M., Xie S., Polekhina G., Wolfe R., Nelson M. (2020). Genetic variation in PEAR1, cardiovascular outcomes and effects of aspirin in a healthy elderly population. Clin Pharmacol Ther.

[37] Lewis J.P., Ryan K., O’Connell J.R., Horenstein R.B., Damcott C.M., Gibson Q. (2013). Genetic variation in PEAR1 is associated with platelet aggregation and cardiovascular outcomes. Circ Cardiovasc Genet.

[38] Xu K., Zheng X., Cai J., Chan N., Shen L., He B. (2020). PEAR1 rs12041331 polymorphisms and the risk of adverse cardiovascular outcomes in patients with acute coronary syndrome and/or percutaneous coronary intervention: a systematic review and meta-analysis. Eur Heart J.

[39] Izzi B., Gianfagna F., Yang W.-Y., Cludts K., De Curtis A., Verhamme P., on behalf of Moli-family I. (2019). Variation of PEAR1 DNA methylation influences platelet and leukocyte function. Clin Epigenetics.

[40] Gresele P. (2015). Subcommittee on Platelet Physiology of the International Society on Thrombosis and Hemostasis. Diagnosis of inherited platelet function disorders: guidance from the SSC of the ISTH. J Thromb Haemost.

[41] Frelinger A.L., Rivera J., Connor D.E., Freson K., Greinacher A. (2021). Consensus recommendations on flow cytometry for the assessment of inherited and acquired disorders of platelet number and function: communication from the ISTH SSC Subcommittee on Platelet Physiology. J Thromb Haemost.

[42] Zivkovic M., Pols-van Veen E., van der Vegte V., Sebastian S.A.E., de Moor A.S., Korporaal S.J.A. (2024). Functional characterization of a nanobody-based glycoprotein VI-specific platelet agonist. Res Pract Thromb Haemost.

