# Supplementary material for: A minimum valency of 4 is required for robust activation of platelets in flow cytometry by multivalent nanobodies to Glycoprotein VI, C-type lectin-like receptor 2 and Platelet Endothelial Aggregation Receptor 1
Source: Res Pract Thromb Haemost. 2025 Sep 24;9(7):103196. doi: 10.1016/j.rpth.2025.103196 (PMC12557594; doi:10.1016/j.rpth.2025.103196)
Supplement: Supplementary figures [file mmc1.pptx]

## Slide 1
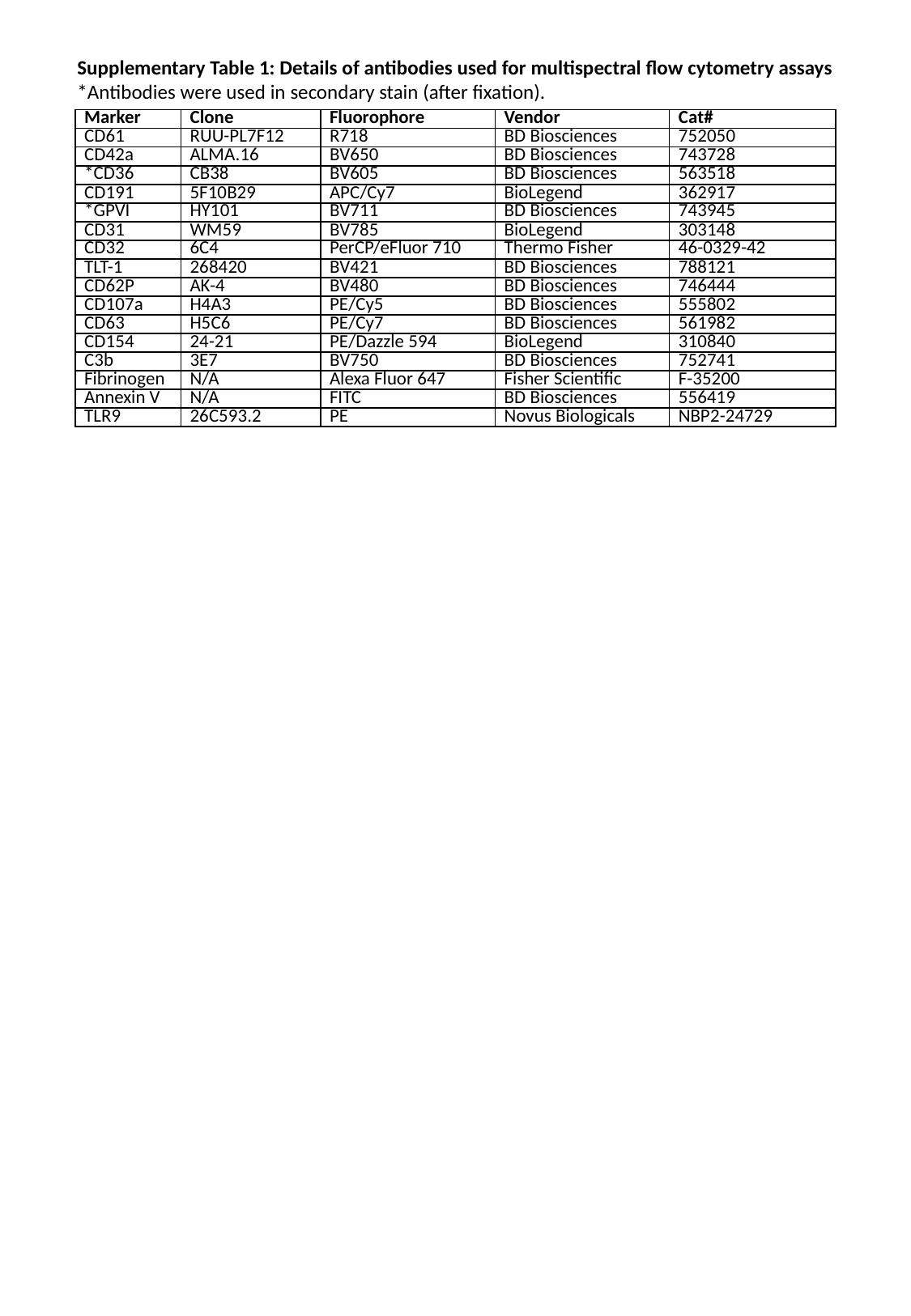

Supplementary Table 1: Details of antibodies used for multispectral flow cytometry assays
*Antibodies were used in secondary stain (after fixation).
| Marker | Clone | Fluorophore | Vendor | Cat# |
| --- | --- | --- | --- | --- |
| CD61 | RUU-PL7F12 | R718 | BD Biosciences | 752050 |
| CD42a | ALMA.16 | BV650 | BD Biosciences | 743728 |
| \*CD36 | CB38 | BV605 | BD Biosciences | 563518 |
| CD191 | 5F10B29 | APC/Cy7 | BioLegend | 362917 |
| \*GPVI | HY101 | BV711 | BD Biosciences | 743945 |
| CD31 | WM59 | BV785 | BioLegend | 303148 |
| CD32 | 6C4 | PerCP/eFluor 710 | Thermo Fisher | 46-0329-42 |
| TLT-1 | 268420 | BV421 | BD Biosciences | 788121 |
| CD62P | AK-4 | BV480 | BD Biosciences | 746444 |
| CD107a | H4A3 | PE/Cy5 | BD Biosciences | 555802 |
| CD63 | H5C6 | PE/Cy7 | BD Biosciences | 561982 |
| CD154 | 24-21 | PE/Dazzle 594 | BioLegend | 310840 |
| C3b | 3E7 | BV750 | BD Biosciences | 752741 |
| Fibrinogen | N/A | Alexa Fluor 647 | Fisher Scientific | F-35200 |
| Annexin V | N/A | FITC | BD Biosciences | 556419 |
| TLR9 | 26C593.2 | PE | Novus Biologicals | NBP2-24729 |

## Slide 2
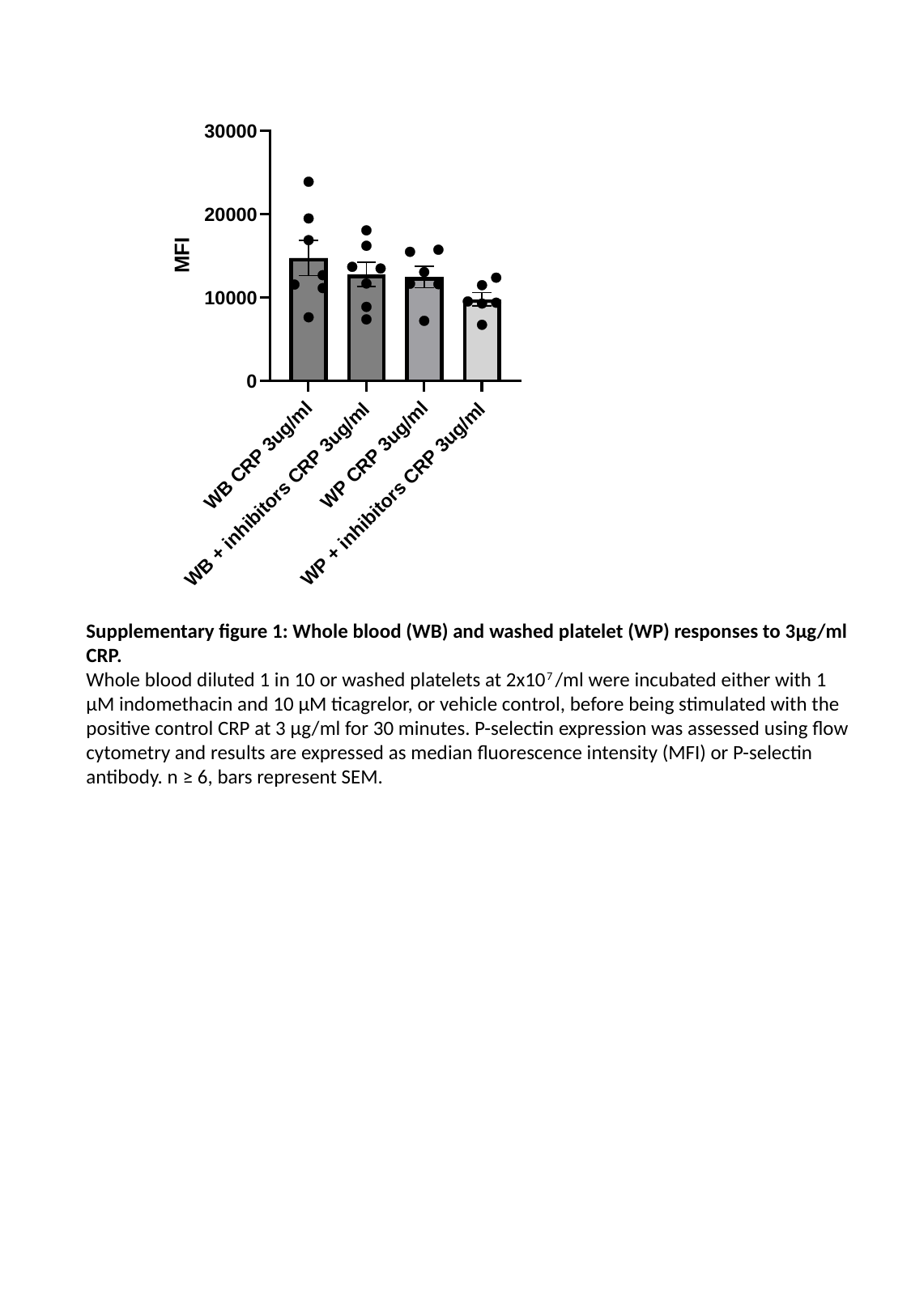

Supplementary figure 1: Whole blood (WB) and washed platelet (WP) responses to 3µg/ml CRP.
Whole blood diluted 1 in 10 or washed platelets at 2x10^7^ /ml were incubated either with 1 µM indomethacin and 10 µM ticagrelor, or vehicle control, before being stimulated with the positive control CRP at 3 µg/ml for 30 minutes. P-selectin expression was assessed using flow cytometry and results are expressed as median fluorescence intensity (MFI) or P-selectin antibody. n ≥ 6, bars represent SEM.

## Slide 3
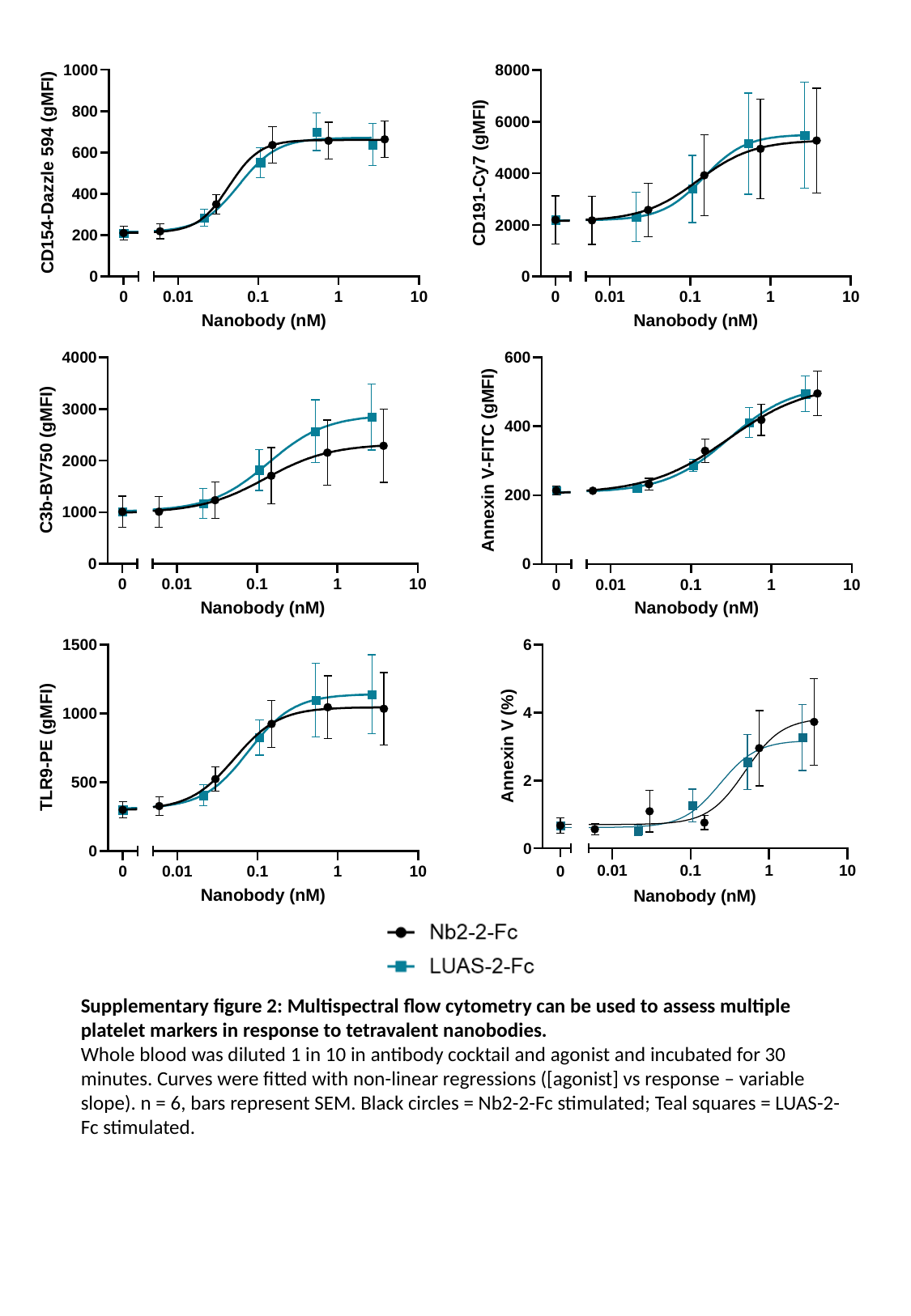

Supplementary figure 2: Multispectral flow cytometry can be used to assess multiple platelet markers in response to tetravalent nanobodies.
Whole blood was diluted 1 in 10 in antibody cocktail and agonist and incubated for 30 minutes. Curves were fitted with non-linear regressions ([agonist] vs response – variable slope). n = 6, bars represent SEM. Black circles = Nb2-2-Fc stimulated; Teal squares = LUAS-2-Fc stimulated.

## Slide 4
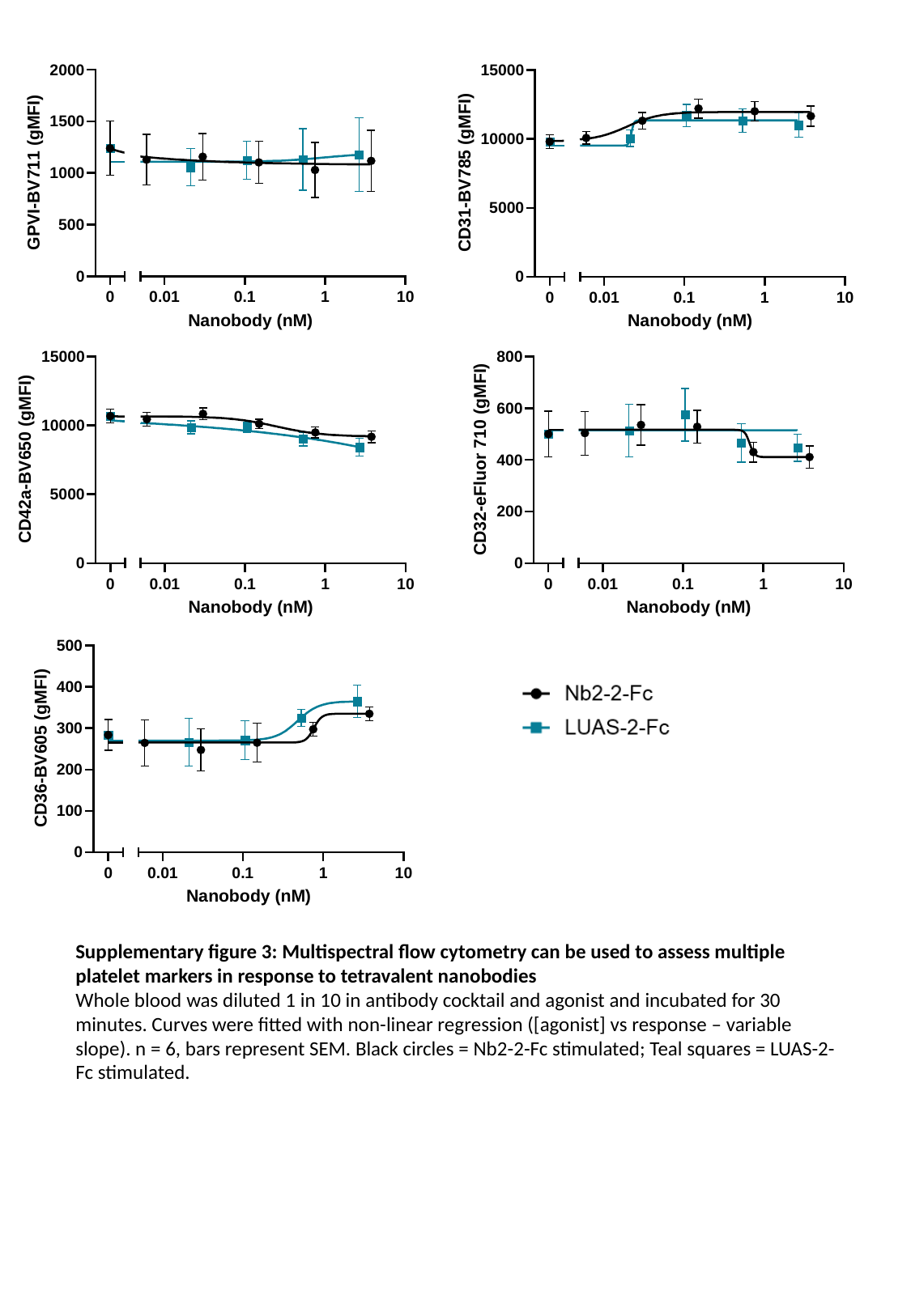

Supplementary figure 3: Multispectral flow cytometry can be used to assess multiple platelet markers in response to tetravalent nanobodies
Whole blood was diluted 1 in 10 in antibody cocktail and agonist and incubated for 30 minutes. Curves were fitted with non-linear regression ([agonist] vs response – variable slope). n = 6, bars represent SEM. Black circles = Nb2-2-Fc stimulated; Teal squares = LUAS-2-Fc stimulated.

## Slide 5
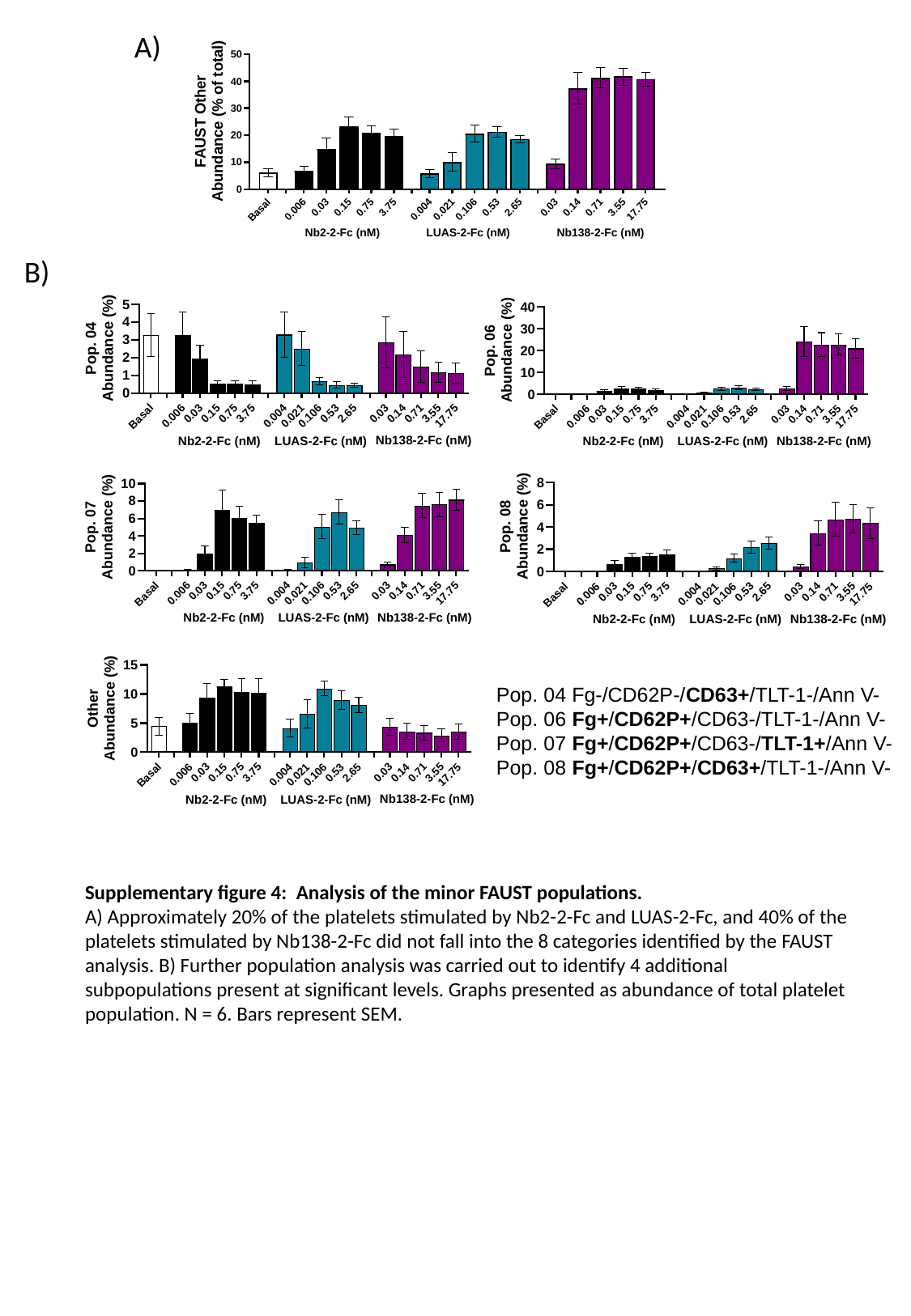

A)
B)
Pop. 04 Fg-/CD62P-/CD63+/TLT-1-/Ann V-
Pop. 06 Fg+/CD62P+/CD63-/TLT-1-/Ann V-
Pop. 07 Fg+/CD62P+/CD63-/TLT-1+/Ann V-
Pop. 08 Fg+/CD62P+/CD63+/TLT-1-/Ann V-
Supplementary figure 4: Analysis of the minor FAUST populations.
A) Approximately 20% of the platelets stimulated by Nb2-2-Fc and LUAS-2-Fc, and 40% of the platelets stimulated by Nb138-2-Fc did not fall into the 8 categories identified by the FAUST analysis. B) Further population analysis was carried out to identify 4 additional subpopulations present at significant levels. Graphs presented as abundance of total platelet population. N = 6. Bars represent SEM.

## Slide 6
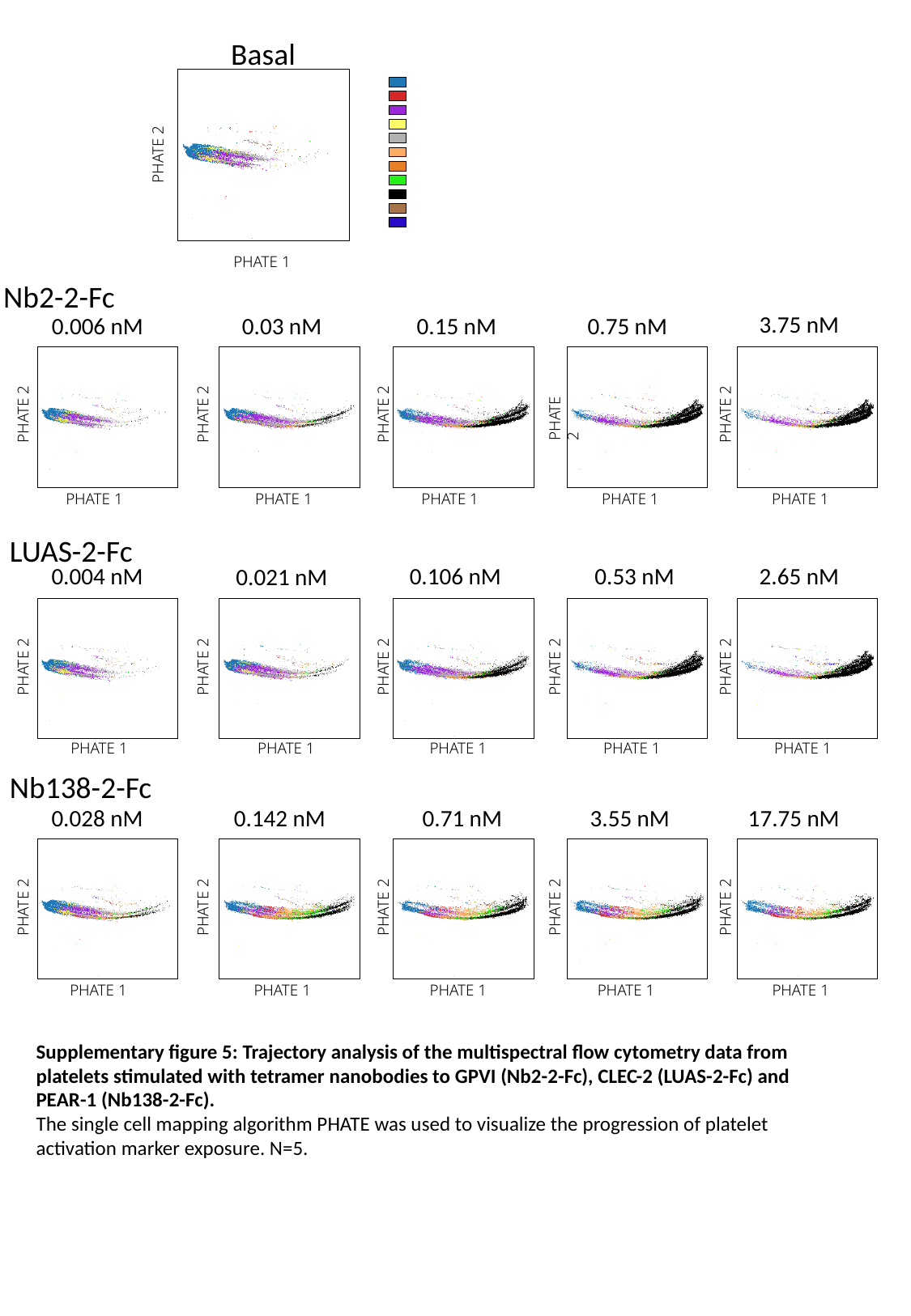

Basal
PHATE 2
PHATE 1
Nb2-2-Fc
3.75 nM
0.006 nM
0.03 nM
0.15 nM
0.75 nM
PHATE 2
PHATE 2
PHATE 2
PHATE 2
PHATE 2
PHATE 1
PHATE 1
PHATE 1
PHATE 1
PHATE 1
LUAS-2-Fc
0.106 nM
0.004 nM
0.53 nM
2.65 nM
0.021 nM
PHATE 2
PHATE 2
PHATE 2
PHATE 2
PHATE 2
PHATE 1
PHATE 1
PHATE 1
PHATE 1
PHATE 1
Nb138-2-Fc
0.028 nM
0.142 nM
0.71 nM
3.55 nM
17.75 nM
PHATE 2
PHATE 2
PHATE 2
PHATE 2
PHATE 2
PHATE 1
PHATE 1
PHATE 1
PHATE 1
PHATE 1
Supplementary figure 5: Trajectory analysis of the multispectral flow cytometry data from platelets stimulated with tetramer nanobodies to GPVI (Nb2-2-Fc), CLEC-2 (LUAS-2-Fc) and PEAR-1 (Nb138-2-Fc).
The single cell mapping algorithm PHATE was used to visualize the progression of platelet activation marker exposure. N=5.

## Slide 7
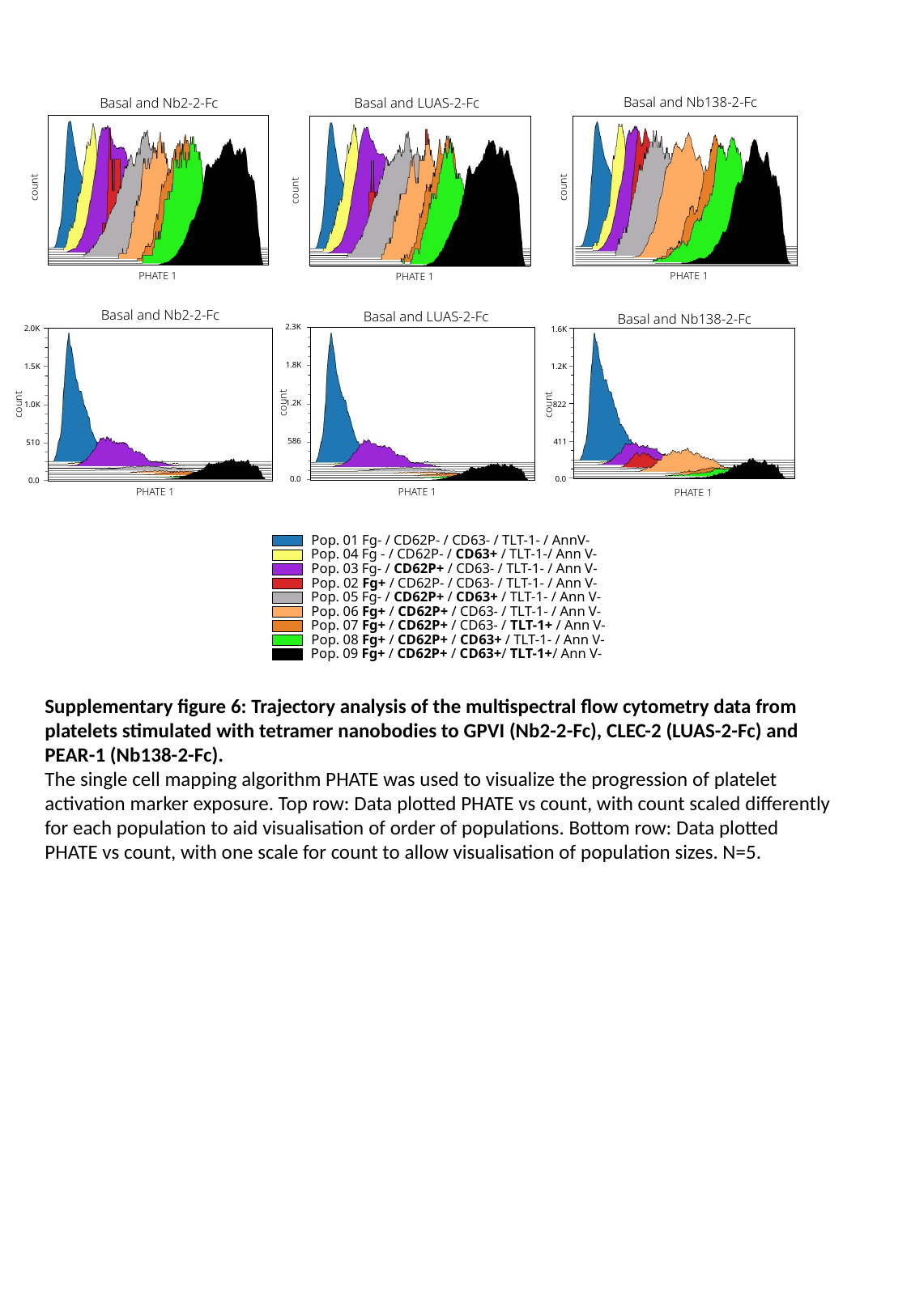

Basal and Nb138-2-Fc
Basal and Nb2-2-Fc
count
PHATE 1
Basal and LUAS-2-Fc
PHATE 1
count
count
PHATE 1
Basal and Nb2-2-Fc
Basal and LUAS-2-Fc
Basal and Nb138-2-Fc
2.3K
1.8K
1.2K
586
0.0
2.0K
1.5K
1.0K
510
0.0
1.6K
1.2K
822
411
0.0
count
count
count
PHATE 1
PHATE 1
PHATE 1
Pop. 01 Fg- / CD62P- / CD63- / TLT-1- / AnnV-
Pop. 04 Fg - / CD62P- / CD63+ / TLT-1-/ Ann V-
Pop. 03 Fg- / CD62P+ / CD63- / TLT-1- / Ann V-
Pop. 02 Fg+ / CD62P- / CD63- / TLT-1- / Ann V-
Pop. 05 Fg- / CD62P+ / CD63+ / TLT-1- / Ann V-
Pop. 06 Fg+ / CD62P+ / CD63- / TLT-1- / Ann V-
Pop. 07 Fg+ / CD62P+ / CD63- / TLT-1+ / Ann V-
Pop. 08 Fg+ / CD62P+ / CD63+ / TLT-1- / Ann V-
Pop. 09 Fg+ / CD62P+ / CD63+/ TLT-1+/ Ann V-
Supplementary figure 6: Trajectory analysis of the multispectral flow cytometry data from platelets stimulated with tetramer nanobodies to GPVI (Nb2-2-Fc), CLEC-2 (LUAS-2-Fc) and PEAR-1 (Nb138-2-Fc).
The single cell mapping algorithm PHATE was used to visualize the progression of platelet activation marker exposure. Top row: Data plotted PHATE vs count, with count scaled differently for each population to aid visualisation of order of populations. Bottom row: Data plotted PHATE vs count, with one scale for count to allow visualisation of population sizes. N=5.
